# A SWOT‐Consensus for CAR‐T in Follicular Lymphoma: Fine Tuning of Patient Journey and Selection

**DOI:** 10.1002/hon.70125

**Published:** 2025-09-23

**Authors:** Monia Marchetti, Paolo Corradini, Luca Arcaini, Stefania Bramanti, Alice Di Rocco, Marco Ladetto, Stefano Luminari, Luigi Rigacci, Pier Luigi Zinzani

**Affiliations:** ^1^ SCDU Ematologia Azienda Ospedaliera SS Antonio e Biagio e Cesare Arrigo Alessandria Italy; ^2^ Divisione di Ematologia Fondazione IRCCS Istituto Nazionale dei Tumori di Milano Cattedra di Ematologia Università degli Studi di Milano Milan Italy; ^3^ Divisione di Ematologia Dipartimento di Medicina Molecolare Università di Pavia Fondazione IRCCS Policlinico San Matteo Pavia Italy; ^4^ IRCCS Humanitas Research Hospital Haematology Milan Italy; ^5^ Dipartimento Medicina Traslazionale e di Precisione Ematologia Università Sapienza di Roma Roma Italy; ^6^ Università del Piemonte Orientale SCDU Ematologia Azienda Ospedaliera SS Antonio e Biagio e Cesare Arrigo Alessandria Italy; ^7^ Ematologia Azienda USL IRCCS Reggio Emilia Reggio Emilia Italy; ^8^ CHIMOMO Department Università di Modena e Reggio Emilia Reggio Emilia Italy; ^9^ UOC Ematologia Fondazione Policlinico Universitario Campus Bio‐Medico e Research Unit of Hematology Department of Medicine and Surgery Università Campus Bio‐Medico Roma Roma Italy; ^10^ IRCCS Azienda Ospedaliero‐Universitaria di Bologna Istituto di Ematologia “Seràgnoli” Bologna Italy; ^11^ Dipartimento di Scienze Mediche e Chirurgiche Università di Bologna Bologna Italy

**Keywords:** CAR‐T therapy, early patient selection, relapsed/refractory follicular lymphoma, third‐line treatment

## Abstract

Over the past 2 decades, advancements in follicular lymphoma (FL) treatment, particularly with anti‐CD20 antibodies, have significantly improved patient survival. However, a subset of FL patients experiences early relapse and progression within 24 months (POD24) after first line treatment, which is a sign of poor prognosis. Current guidelines recommend various second‐line treatments, but there is no consensus on an optimal treatment sequence for relapsed/refractory (r/r) FL. Moreover, despite available treatments, reduced survival after second‐line therapies and diminishing responses with each relapse highlight the unmet need for more effective options. Chimeric antigen receptor T‐cell (CAR‐T) therapy has emerged as a promising treatment for r/r FL beyond 2^nd^ line therapy, with three FDA/EMA‐approved therapies (axicabtagene ciloleucel, tisagenlecleucel, and lisocabtagene maraleucel) showing high efficacy and manageable side effects. However, challenges remain in determining which patients will benefit most from CAR‐T, especially given its high cost, safety concerns, and logistical barriers. A consensus study was conducted to guide CAR‐T patient selection and treatment sequencing for patients in 3rd line or beyond. Key findings suggest that younger patients, those with high disease burden or poor first‐line responses, should be prioritized for CAR‐T. Additionally, CAR‐T is recommended as a third‐line option for patients with POD24, double refractoriness (failure to respond to two subsequent lines of immunochemotherapy), or early autologous stem cell transplant failure. The study underscores the importance of early assessment of treatment response, careful second‐line therapy selection, and patient adherence to ensure optimal outcomes. The results, based on expert consensus, support CAR‐T therapy as a viable option for r/r FL patients, offering hope for durable remissions in this challenging cohort.

## 1 Introduction

Follicular lymphoma (FL) is one of the most common indolent non‐Hodgkin lymphomas (NHL) in Western countries, with an incidence of approximately 3.5 new cases per 100,000 individuals annually in the United States [1, 2, 3]. Over the last 20 years, the prognosis for FL has significantly improved, largely due to the introduction of anti‐CD20 monoclonal antibodies such as rituximab. This advancement has led to a 10‐year overall survival (OS) rate of approximately 80% in France and 75% in the United States [4, 5, 6].

First‐line treatments for FL depend on the disease stage and tumor burden. Treatment for early‐stage or low‐tumor‐burden FL typically includes radiotherapy and/or anti‐CD20 antibody therapy [7, 8, 9, 10]. In contrast, advanced‐stage disease generally requires immunochemotherapy [11, 12]. The PRIMA trial showed that maintenance rituximab after R‐CHOP or R‐CVP significantly enhances progression‐free survival (PFS) [13], and the FOLL05 study confirmed the superiority of R‐CHOP over R‐CVP [14, 15]. More recently, bendamustine combined with rituximab has emerged as the preferred first‐line regimen, supported by findings from the StiL NHL1 and BRIGHT trials [16, 17]. Other trials like RELEVANCE [18] and GALLIUM [19, 20] explored alternative front‐line approaches, including the use of obinutuzumab in place of rituximab and the incorporation of lenalidomide.

Despite these improvements, around 20% of patients experience early disease progression within 24 months of initial therapy (POD24), which is associated with significantly poorer outcomes. These patients have a 5‐year OS of less than 50%, compared to more than 90% in those without early progression [21]. Several second‐line options are available [12, 22], such as rituximab, lenalidomide‐rituximab, bendamustine‐obinutuzumab, and other chemoimmunotherapy regimens [23]. According to NCCN guidelines, patients with symptomatic or high‐tumor‐burden disease should receive non–cross‐resistant anti‐CD20 antibody combinations or lenalidomide‐rituximab [22].

For patients who relapse early following rituximab‐based therapies, autologous stem cell transplantation (ASCT) remains an option. While early ASCT can improve 5‐year OS [24], the FLAZ12 study reported no significant survival advantage compared to radioimmunotherapy and found higher toxicity in the ASCT arm [25].

Third‐line treatment strategies typically involve novel therapies or drug combinations not previously used. Targeted agents like tazemetostat, an EZH2 inhibitor [26], and zanubrutinib, a BTK inhibitor used with obinutuzumab [27], have recently expanded the therapeutic landscape. However, treatment efficacy tends to decline with each additional line of therapy, and long‐term quality of life remains challenged by cumulative toxicity [6, 28, 29, 30].

Chimeric antigen receptor T‐cell (CAR‐T) therapy has revolutionized the treatment of hematologic malignancies, including acute lymphoblastic leukemia, multiple myeloma, and lymphomas [31]. In the context of relapsed/refractory (r/r) FL, CAR‐T therapies such as axicabtagene ciloleucel (axi‐cel, ZUMA‐5) [32, 33, 34], tisagenlecleucel (tisa‐cel, ELARA) [30, 35], and lisocabtagene maraleucel (liso‐cel, TRANSCEND‐FL) [36] have shown high response rates, durable remissions, and manageable safety profiles.

Bispecific antibodies (BsAbs), including mosunetuzumab (CD20/CD3), have also shown clinical activity even in patients who previously received CAR‐T, and are now approved for use after two prior therapies [37, 38]. Other investigational agents include epcoritamab [39] and odronextamab [40]. Though both CAR‐T and BsAb therapies tend to outperform conventional chemotherapy [31], their precise role in the treatment sequence is still debated [41]. Real‐world barriers such as toxicity, cost, access, and impaired T‐cell fitness due to prior treatments remain significant [4, 41, 42].

To better define the role of CAR‐T therapy and address these uncertainties, a GRADE‐based consensus initiative was undertaken to establish eligibility criteria, guide sequencing strategies, and inform clinical pathways in third‐line treatment.

## 2 Methods

The methodology was grounded in the GRADE framework, which involves formulating clinical questions using the PICO (Population, Intervention, Comparison, Outcome) format. An initial virtual meeting was held in January 2024 with a Core Panel of eight national key opinion leaders. They developed clinical questions and proposed outcomes, ranking their relevance on a 1–7 Likert scale. The top three outcomes with the highest rankings (score of 7) were selected. Similarly, specific patient subgroups were identified and ranked by relevance; those scoring 5, 6, or 7 by more than 50% of panelists were included.

For each PICO‐formulated question, a non‐systematic literature review was performed using EMBASE, limited to meta‐analyses. In addition, a SWOT analysis was applied to each question: clinical benefits were categorized as Strengths, adverse clinical effects as Weaknesses, opportunities in underserved subgroups as Opportunities, and non‐clinical barriers as Threats.

The output of these analyses led to the development of Good Practice Statements (GPS), Research Statements (RS), and Remarks. GPS are actionable, evidence‐informed recommendations with an anticipated net benefit. RS reflect weaker evidence and uncertain benefit. Remarks are non‐prescriptive statements supporting a GPS by clarifying application methods, relevant subgroups, or definitions [43].

Expert agreement with these statements was evaluated through the Delphi method, an indirect, anonymous, and iterative approach for achieving expert consensus, commonly used in the context of disease management and therapeutic strategies [44]. A separate External Panel of 12 senior hematologists participated in a Delphi survey, ranking agreement on a 1–9 Likert scale (1 = total disagreement; 9 = total agreement). A statement was considered appropriate if the median score was ≥ 7 and consensus was reached, following the RAND/UCLA Appropriateness Method User's Manual [45].

**FIGURE 1:**
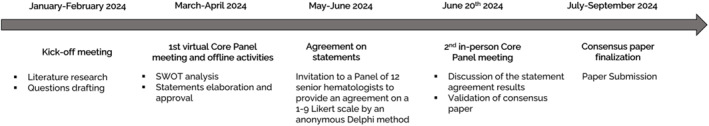
Steps of the consensus project.

The final Delphi results were reviewed and discussed during an in‐person Core Panel meeting on June 20, 2024, during which the structure and contents of the consensus document were finalized (Figure 1).

## 3 Results

The following *Clinical Questions* were elaborated by the Panel:

*Q1*: Which FL patients should receive specific pre‐assessment during frontline therapy in order to optimize their eventual subsequent journey‐to‐CAR‐T, possibly optimizing CAR‐T therapy outcomes while granting the optimal sequencing of therapies?

*Q2*: Which 2nd‐line FL patients should be pre‐assessed and/or referred before starting their 2^nd^‐line therapy in order to optimize timing and efficiency of their journey‐to‐CART at eventual next relapse and possibly optimize CAR‐T therapy outcomes while granting the optimal sequencing of therapies?

*Q3*: Which 3rd‐line FL patients should receive CAR‐T rather than bispecific antibodies or other reimbursed therapies?

Question 3 was subsequently divided into two sub‐questions, to better address exclusion (question 3A) or inclusion (question 3B) criteria for recruiting eligible patients for CAR‐T.

### 3.1 Outcome and Subgroup Selection

#### 3.1.1 Question 1

##### 3.1.1.1 Outcomes and Subgroups

Which outcomes and which clinical and non‐clinical factors are most relevant to be evaluated during first‐line treatment to identify eligible patients for future CAR‐T therapy?

**FIGURE 2:**
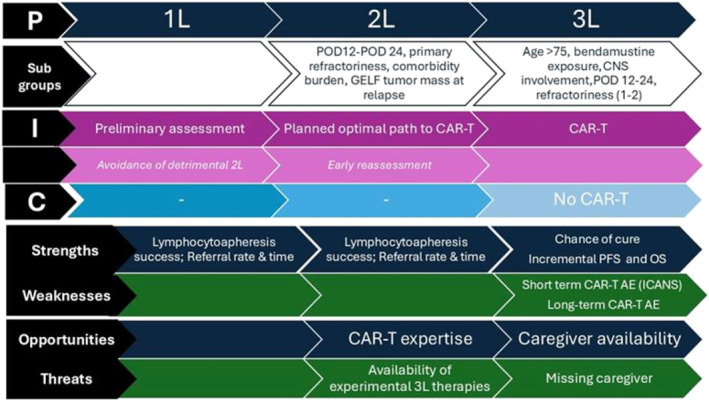
Summary of the main results of the study.

Proposed items and results are reported in Supporting Information S1: Figure 1 and 2.

Among outcomes, the choice of second‐line therapy and referring the patient to the reference CAR‐T center were considered most relevant. Regarding subgroups, age, disease burden, FLIPI at diagnosis, expertise, and logistics were considered the most relevant clinical and non‐clinical factors.

#### 3.1.2 Question 2

##### 3.1.2.1 Outcomes and Subgroups

Which outcomes and clinical and non‐clinical factors are most relevant to be early evaluated to identify eligible 2^nd^‐line patients for the possible use of CART as third‐line therapy?

Proposed items and results are reported in Supporting Information S1: Figure 3 and 4.

Among outcomes, the most frequently voted as most important were ensuring optimal lymphocytoapheresis and avoiding detrimental drugs for 2^nd^‐line treatment. Referral time, lead time to CAR‐T, the ability to grant optimal sequencing, to plan early reassessment, and to perform pre‐workup of inclusion and exclusion criteria for CAR‐T were also voted as important.

Among subgroups, the most relevant clinical factors were POD12 at 1^st^ relapse, POD24 at 1st relapse, refractoriness to first‐line therapy, comorbidity burden, and GELF tumor mass at relapse.

#### 3.1.3 Question 3A

##### 3.1.3.1 Outcomes and Subgroups

Which outcomes and which clinical and non‐clinical factors are most relevant to be evaluated to identify patients not eligible for CART as third‐line therapy (although treatment eligible)?

Proposed items and results are reported in Supporting Information S1: Figures 5 and 6.

Among outcomes, long‐term toxicities (cytopenia and infections) and achievement of plateau or cure were ranked as most important. A high ICANS score and PFS were also considered relevant.

Among clinical factors, previous bendamustine use, frailty, and age > 75 years were ranked as most important, as well as CNS involvement (or other ELARA exclusion criteria) and symptomatic relapse. Previous recurrent/severe infections and comorbidities, absence of GELF criteria, or presence of contraindications to steroids use were valued as important by a few participants. Among non‐clinical factors, caregiver availability was ranked as most important.

#### 3.1.4 Question 3B

##### 3.1.4.1 Outcomes and Subgroups

Which outcomes and clinical and non‐clinical factors are most relevant to be evaluated to identify patients eligible for CART as third‐line therapy?

Proposed items and results are reported in Supporting Information S1: Figures 7 and 8.

Among outcomes, OS, PFS, and plateau of cure were ranked as most important. Among clinical factors, POD12/24, refractoriness, and double CIT refractoriness were ranked as most important. Failure of a previous ASCT was also considered an important factor to be taken into consideration in the assessment of CAR‐T eligibility. Among non‐clinical factors, only caregiver availability was ranked as important.

A summary of outcomes and subgroups selected based on ranking results is reported in Table 1.

**TABLE 1:** Summary of selected outcomes and subgroup after ranking.

Question	Population	Intervention	Outcome	Clinical
				subgroups
1	FL patients on 1^st^ line therapy	CAR‐T pre‐assessment	Optimization of 2^nd^ line therapy choice Timely referral to CART center	Age Burden of disease FLIPI at diagnosis
2	FL patients candidate to 2^nd^ line therapy	Potential CAR‐T candidates tracking	Avoidance of detrimental 2^nd^ line therapies Grant optimal lymphocytoapheresis Referral time Lead time to CAR‐T Grant optimal sequencing Plan early reassessment Perform pre‐work‐up of inclusion and exclusion criteria to CART	POD12 POD24 Primary refractory Comorbidity burden GELF tumor mass at relapse
3A	FL patients candidate to 3^rd^ line therapy	Exclusion of patients not deemed to undergo‐CAR‐T	Plateau or cure Long‐term AE ICANS PFS	Bendamustine exposure 9–12 months Frailty Age over 75 years CNS involvement (or other ELARA exclusion criteria) Symptomatic relapse.
3B		Inclusion of patients candidate to CAR‐T	OS PFS Plateau or cure	POD 12–24 Refractoriness Double CIT refractoriness Failure of a previous ASCT

### 3.2 SWOT Analysis

Each PICO's clinical and non‐clinical outcomes were further analyzed using the SWOT framework. According to such analysis, favorable clinical outcomes were listed as Strengths of the proposed interventions, while non‐favorable clinical outcomes were listed as Weaknesses. Similarly, favorable non‐clinical outcomes were listed as Opportunities and non‐favorable non‐clinical outcomes as Threats. The SWOT framework supported the Core Panel task of elaborating transparent statements. Strengths, Weaknesses, Opportunities and Threats included in the different statements were listed in Table 2.

**TABLE 2:** SWOT analysis (statements code reported).

Strenght	Weakness	Opportunities	Threats
Amelioration of the dismal OS and PFS by CAR‐T in the 3L. The highest net benefit detected in refractory disease (especially double refractory patients) and in patients with early relapse (POD24). Net benefit assured also in patients with prior ASCT, and across different FLIPI‐at‐relapse or MTV‐at‐relapse classes.	Lack of plateau in PFS curves, which however was scored less important than the overall survival outcome itself. Net benefit (OS and PFS vs. AE) versus non‐ CART (less intensive) therapies (or WW) are less straightforward in 3L patients with an expected better outcome, namely those with late (possibly localized or asymptomatic) relapses Expected toxicities higher in elderly patients (vs. life expectancy gain) or psychiatric and neurologic disturbances	One‐shot therapy. Therapeutic sequencing optimized (bispecific antibodies feasible at further failure)	Hospital stay cost Availability (not off‐the‐shelf) Access (referral to hub centers; center capacity) Caregiver support
2.1 3.1	3.2 3.4 3.5 2.1.1		2.1.3 3.4.2
Higher net benefit in timely identified young patients with high risk and/or high burden disease and/or suboptimal response or early relapse.	Expected lymphocyte apheresis failure in patients recently exposed to bendamustine.	Expertise CAR‐T pathways	Timely referral
1.1 1.2 2.3	2.2 3.3	1.1.1 2.1.2	3.1.2 2.3
	Net benefit of CAR‐T in patients with relevant comorbidities still to be confirmed definitely. Net benefit of CAR‐T in patients with CNS involvement still to be confirmed definitely.		
	3.6		

### 3.3 Statement Elaboration and Approval (Delphi)

A total of 20 statements were elaborated by the core Panel, and classified as GPS, RS or R, according to the definitions reported above (Table 3).

**TABLE 3:** Statements elaborated by the core Panel for the 3 clinical questions.

N.	Statement	ID	Question
1	In order to improve the dismal survival of patients who failed two prior lines and show refractory disease and/or early relapse, CAR‐T therapy is strongly recommended, irrespectively of prior ASCT and of FLIPI or metabolic tumor volume at relapse	GPR 3.1	3
2	While no single definition of early relapse has been established, progression of disease within 24 months of initial treatment (POD24) is widely accepted as a critical adverse prognostic factor	R 3.1.1	3
3	Patients with NHL in need of CAR‐T therapy should be granted timely access to this treatment irrespectively of the capacity of local centers. Therefore, a regional or countrywide flow of patients to networked CAR‐T centers is desirable to achieve this goal	R 3.1.2	3
4	Patients with late relapses deserve a careful assessment of the available therapeutic options (included watchful wait)	GPR 3.2	3
5	Those patients who have been exposed to bendamustine in the last 6 months should better be considered for treatments alternative to CAR‐T	GPR 3.3	3
6	Indirect evidence suggests that patients having been exposed to bendamustine in the last 7–9 months might face a detrimental outcome, therefore a more careful assessment of the best therapeutic choice should be completed in such persons	R 3.3.1	3
7	In order to avoid unacceptable toxicity of immunotherapies, particularly long‐term cytopenias and infections and neurologic toxicity, very elderly patients should be carefully screened with validated frailty tools before confirming their eligibility	GPR 3.4	3
8	The decision to undergo CAR‐T should be carefully evaluated in the rare patients with psychiatric disturbances that absolutely contraindicate steroids	R 3.4.1	3
9	The absence of family support/caregiver should be considered a relevant hurdle to the assignment of patients to cellular immunotherapy with CAR‐T	R 3.4.2	3
10	The net benefit of third‐line immunotherapies in patients with a history of severe or recurrent infections (with/without severe hypogammaglobulinemia) should be specifically investigated by specific studies	RS 3.5	3
11	The net benefit of third‐line CAR‐T in patients with specific comorbidities, which excluded patients from registrative trials, should be specifically investigated by specific studies	RS 3.6	3
12	In order to foster an efficient journey‐to‐CAR‐T, preliminary assessment of CAR‐T eligibility items is recommended in FL patients who proved refractory to frontline therapy, incurred an early relapse (POD24), or showed a high disease burden at relapse (GELF mass criteria or metabolic tumor burden), provided that they are free of relevant comorbidities	GPR 2.1	2
13	The most relevant comorbidities to be checked include neurologic ones, in particular seizures	R 2.1.1	2
14	An appropriate expertise needs to be developed at each center caring for FL in order to support each phase of the CAR‐T eligibility assessment and referral process	R 2.1.2	2
15	The physicians caring for potential CAR‐T candidates should check patient willingness to adhere to an eventual CAR‐T process and to verify the availability of an appropriate caregiver	R 2.1.3	2
16	Preliminarily identified potential candidates to subsequent CAR‐T therapy (see recommendation 2.1) should not be exposed to second‐line treatments which might negatively interfere with an optimal therapeutic sequencing	GPR 2.2	2
17	In order to foster an efficient journey to CAR‐T, early assessment of response is recommended in FL patients (see recommendation 2.1)	GPR 2.3	2
18	In order to timely implement a possible use of CAR‐T therapy in subsequent lines, hematology centers are recommended to settle a specific “CAR‐T pathway” starting since the diagnosis in younger FL patients and in those showing a high disease burden (FLIPI/m7FLIPI, metabolic tumor burden)	GPR 1.1	1
19	Both clinical and organizational know‐how need to be built up at each hematology center in order to support the CAR‐T pathway	R 1.1.1	1
20	A CAR‐T focused choice of second‐line treatment is recommended in patients diagnosed at a young age, with a high disease burden, or with suboptimal response after frontline therapy	GPR 1.2	1

Abbreviations: GPR, Good Practice Recommendation; R, Remark; RS, Research Statement.

The statements were subsequently proposed to the Expert Panel to assess the degree of agreement on a 9‐point Likert scale. A total of 12 experts participated in the voting phase. All the proposed statements met the predefined cut‐off for consensus definition (median scores ≥ 7) after the first round of voting. Results for each statement (divided by question) are reported below and in Supporting Information S1: Table 1.

#### 3.3.1 Question 3

##### 3.3.1.1 Statement 1—GPS 3.1 (Median: 7.0)

###### 3.3.1.1.1 Related Evidence/Comments

Patients with FL often respond well to early treatments, but remission shortens after ≥ 2 prior LoT. Few r/r FL patients achieve CR, and about 1/3 die within 24 months. Shorter median PFS with more prior LoT suggests suboptimal treatment durability, leaving patients underserved in the US and Europe [46].

CAR‐T therapies have shown benefits in aggressive lymphomas. In the ZUMA trial, axi‐cel demonstrated high, durable responses with manageable safety in r/r indolent NHL, even among high‐risk patients [32]. Similarly, ELARA data show that tisa‐cel is effective in heavily pretreated r/r FL, with high CRRs and ORRs regardless of risk factors [30, 35]. The durable responses and manageable safety from ZUMA‐5 and ELARA suggest CAR‐T therapy could significantly impact outcomes.

##### 3.3.1.2 Statement 2—R 3.1.1 (Median: 9.0)

###### 3.3.1.2.1 Related Evidence/Comments

Several studies show that 20%–30% of patients experience POD24, with a 5‐year OS of 50% compared to 90% in those without early progression [47, 48]. Observational studies and trials confirm poor outcomes for POD24 FL patients. A pooled analysis of 13 trials (> 5000 FL patients) identified male sex, poor performance status, high FLIPI score, and elevated β2‐microglobulin as POD24 risk factors. This validated POD24 as a strong predictor of poor survival and highlighted clinical factors aiding prognostic model development [21].

##### 3.3.1.3 Statement 3—R 3.1.2 (Median: 8.0)

###### 3.3.1.3.1 Related Evidence/Comments

Timely treatment is crucial for lymphoma patients. An Italian analysis of DLBCL patients in 2020 found that 140 were approved for CAR‐T therapy, 120 underwent leukapheresis, and 110 received treatment (37% of eligible patients under AIFA criteria) with a median wait time of 63 days. Barriers to access include patient identification, referral, funding approval, and therapy delivery [49]. Proposed solutions involve harmonized CAR‐T referral networks, better regional coordination, and national planning to optimize specialized CAR‐T centers [49].

##### 3.3.1.4 Statement 4—GPS 3.2 (Median: 8.0)

###### 3.3.1.4.1 Related Evidence/Comments

According to the most recent clinical practice recommendations issued by the American Society of Transplantation and Cellular Therapy and the European Society of Blood and Marrow Transplantation, CAR‐T is recommended to be considered for patients who experience late relapse and do not achieve CR or PR after second or subsequent line of therapy [50].

##### 3.3.1.5 Statement 5—GPS 3.3 (Median 7.0).

##### 3.3.1.6 Statement 6—R 3.3.1 (Median 8.0)

###### 3.3.1.6.1 Related Evidence/Comments

Since CAR‐T cells come from autologous T‐lymphocytes, their composition depends on T‐cell number and fitness. Bendamustine's lymphotoxic effects may hinder CAR‐T production, leading consensus guidelines to advise against its use in CAR‐T candidates. While data in r/r FL are lacking, a study in R/R LBCL found that bendamustine before CAR‐T negatively impacted T‐cell numbers, expansion, and outcomes, especially with recent exposure (< 9 months) [42].

##### 3.3.1.7 Statement 7—GPS 3.4 (Median 8.5)

###### 3.3.1.7.1 RelatedEvidence/Comments

CAR‐T in elderly patients raises concerns about comorbidities, toxicity, and survival. While some studies suggest higher neurotoxicity and NRM in those ≥ 65, response rates and PFS remain comparable or better than in younger patients [51]. A key challenge is the lack of reliable tools to predict toxicity and prognosis [52]. The geriatric assessment (GA) evaluates frailty by considering health domains like physical function, cognition, and social support [53]. GA has improved patient selection for transplantation and could help optimize CAR‐T in older adults [52, 54].

##### 3.3.1.8 Statement 8—R 3.4.1 (Median 9.0)

###### 3.3.1.8.1 Related Evidence/Comments

Corticosteroids help manage severe CAR‐T toxicities by reducing immune cell proliferation and cytokine production. However, studies link higher cumulative doses and prolonged early use to early progression and shorter survival in large B‐cell lymphoma. This suggests corticosteroids should be used minimally, for the shortest duration, and delayed, when possible, to optimize CAR‐T outcomes [55].

##### 3.3.1.9 Statement 9—R 3.4.2 (Median 8.0)

###### 3.3.1.9.1 Related Evidence/Comments

Before CAR‐T, patients often experience strong emotions, viewing it as a last hope for remission. Unique complications like cytokine release syndrome, along with physical pain and a desire for normalcy, add to emotional distress [56]. Family and caregiver support is essential, typically recommended for 30 days post‐infusion, with travel support for up to 60 days [23].

##### 3.3.1.10 Statement 10—RS 3.5 (Median 8.0)

###### 3.3.1.10.1 Related Evidence/Comments

CAR‐T shows promise for aggressive NHL but comes with severe toxicities like CRS, HLH, ICANS, cytopenia, and infections. A meta‐analysis of 15 trials (1364 patients) found higher CRS and ICANS rates with axi‐cel, while liso‐cel had more severe neutropenia. Severe infections were more common with axi‐cel, but febrile neutropenia rates were similar across products. Understanding these toxicities could enable patient‐tailored therapy and early intervention [57].

##### 3.3.1.11 Statement 11—RS 3.6 (Median 8.0)

###### 3.3.1.11.1 Related Evidence/Comments

CAR‐T therapy is effective for relapsed/refractory (R/R) large B‐cell lymphoma (LBCL), but patients with CNS lymphoma were largely excluded from trials. A recent meta‐analysis showed that CAR‐T in CNS LBCL patients is as safe and effective as in those without CNS involvement [58]. Similar studies in r/r FL patients with comorbidities are needed to assess CAR‐T's benefits in these cases.

#### 3.3.2 Question 2

##### 3.3.2.1 Statement 12—GPS 2.1 (Median 9.0)

###### 3.3.2.1.1 Related Evidence/Comments

Although CAR‐T should be considered a treatment option for patients who do not achieve CR or PR after second or subsequent LoT, a preliminary assessment of CAR‐T eligibility before starting second LoT might allow early identification of patients who might benefit of CAR‐T in the subsequent LoT, possibly avoiding treatments which might negatively interfere with an optimal therapeutic sequencing (see recommendation 2.2).

##### 3.3.2.2 Statement 13—R 2.1.1 (Median 8.0)

###### 3.3.2.2.1 RelatedEvidence/Comments

Neurological toxicity in CAR‐T recipients, now termed ICANS, is the second most common adverse event, with incidence rates ranging from 12% to 55% [59]. Pre‐existing neurological comorbidities, as well as factors like ALL, tumor burden, meningeal involvement, and prior CNS therapies, may increase the risk of ICANS [60].

##### 3.3.2.3 Statement 14—R 2.1.2 (Median 8.0)

###### 3.3.2.3.1 RelatedEvidence/Comments

Educational programs and materials for hematologists on CAR‐T eligibility, treatment pathways, and outcomes are crucial for improving patient identification and referral. These initiatives should be collaboratively developed by institutions, clinical centers, the Italian Society of Hematology, patient groups, and pharmaceutical manufacturers [49].

##### 3.3.2.4 Statement 15—R 2.1.3 (Median 8.5)

###### 3.3.2.4.1 Related Evidence/Comments

Patients eligible for CAR‐T should be informed about the benefits, risks, and the need for dedicated caregiver support. Distance from the treatment center may pose barriers to recruitment due to economic or cultural factors, and some patients may prefer outpatient care over in‐hospital treatment.

##### 3.3.2.5 Statement 16—GPS 2.2 (Median 9.0)

###### 3.3.2.5.1 Related Evidence/Comments

After 3 years in ZUMA‐5, axi‐cel showed durable responses with few relapses beyond 2 years and manageable safety in R/R patients. However, assessments of baseline variables, including prior bendamustine use and elevated TMTV, suggested that these factors may impact durable remissions in FL patients [34].

##### 3.3.2.6 Statement 17—GPS 2.3 (Median 7.0)

###### 3.3.2.6.1 Related Evidence/Comments

Early response assessment in FL patients is crucial when considering CAR‐T as a third‐line option. It helps identify patients who will benefit most while avoiding treatment delays. Recognizing early treatment failure allows a transition to second‐line therapies, with CAR‐T being a viable third‐line choice if needed. Timely CAR‐T initiation after therapy failure is linked to better long‐term outcomes, including higher OS and PFS [34].

#### 3.3.3 Question 1

##### 3.3.3.1 Statement 18—GPS 1.1 (Median 7.0)

###### 3.3.3.1.1 Related Evidence/Comments

CAR‐T offers therapeutic efficacy but presents challenges in symptom management, rehabilitation, and psychological care. Patient‐centered care is essential to improve healthcare access and quality [56]. Unlike conventional treatments, CAR‐T involves complex processes, adverse effects like CRS and ICANS, and emotional stress. Addressing its physical and psychological impact is crucial, and effective communication and management are highly valued by patients and caregivers. Further research is needed to reduce burdens and develop self‐management programs [56].

##### 3.3.3.2 Statement 19—R 1.1.1 (Median 7.5)

###### 3.3.3.2.1 Related Evidence/Comments

As immune effector cells (IECs) are increasingly used in commercial and research settings, healthcare organizations must develop procedures to safely administer this therapy. The Foundation for the Accreditation of Cellular Therapy (FACT) has established standards for IEC use, including CAR‐T cells, covering patient selection, cell collection, therapy, toxicity management, and long‐term follow‐up. FACT accreditation improves program quality, ensuring compliance and enabling clinical trial participation and certification for administering commercial IEC therapies [61]. Given IEC therapy's distinct toxicities, a strong procedure for identifying eligible patients is crucial, and personnel education is key to preventing errors and managing hematologic toxicities.

##### 3.3.3.3 Statement 20—GPS 1.2 (Median 8.0)

###### 3.3.3.3.1 Related Evidence/Comments

Young patients with follicular lymphoma, especially those with disease progression after first‐line therapies, may tolerate intensive treatments like CAR‐T better. CAR‐T offers long‐term benefits for these patients, potentially improving life expectancy and quality of life. CAR‐T provides a more targeted option than chemotherapy in cases of high disease burden. After suboptimal responses to frontline therapy, CAR‐T offers a cutting‐edge alternative with the potential for deeper, longer‐lasting responses, particularly in younger patients with better tolerance.

A summary of main results is reported in Figure 2.

## 4 Discussion

The SCHOLAR‐5 study, a retrospective analysis of relapsed/refractory (r/r) follicular lymphoma (FL) patients, revealed no clear standard of care and showed that response rates, quality, and duration of response diminish with each line of therapy (LoT). Progression‐free survival (PFS) dropped from 16.8% at the third LoT to 7.9% at the fifth or later LoT [62]. Similarly, the LEO CReWE study found that high FLIPI scores and alkylator‐refractory disease were associated with shorter PFS, highlighting the unmet need for patients requiring therapy beyond second line [64].

Recently approved CAR‐T therapies have shown efficacy in this setting and are being further evaluated in routine care [63]. CAR‐T is now considered standard for patients with progression of disease within 24 months (POD24) who fail to achieve complete or partial remission after second or later LoTs, as well as for those who relapse after autologous hematopoietic cell transplantation (auto‐HCT) or have late relapses [50]. Both tisa‐cel and axi‐cel administered as 3L treatments have demonstrated durable remissions with 3‐year follow‐up [34, 35], whereas liso‐cel demonstrated efficacy and safety also in 2L patients with high‐risk disease features (POD24 from diagnosis and double refractoriness) [36].

CAR‐T represents a valid option for r/r FL patients beyond second‐line therapy, but timely and accurate identification of eligible candidates is critical to avoid prior interventions that might compromise CAR‐T effectiveness. Early identification remains challenging due to the absence of formal criteria for CAR‐T in earlier LoTs. However, younger patients with high tumor burden or suboptimal responses should be closely monitored (Statements 18, 20), and expert centers are essential to guide the patient journey (Statement 19).

For second‐line therapy, early response assessment is crucial, particularly in refractory or high‐burden patients. Initial CAR‐T eligibility evaluation—including comorbidities—is advised (Statements 12, 13). Education for haemato‐oncologists is key to improving early identification [49], and patient readiness and caregiver support should also be considered (Statement 15). Bendamustine should be avoided in second‐line therapy, as it may impair CAR‐T response (Statement 16).

In third‐line settings, bendamustine history, age (Statement 7), psychiatric health (Statement 8), infection risk (Statement 10), POD24 (Statement 2), double refractoriness (Statement 1), and failed auto‐HCT (Statement 9) must be assessed.

A structured CAR‐T pathway, supported by early assessments and shared expertise, is essential for optimizing outcomes.

## 5 Conclusion

CAR‐T therapy is an effective treatment for FL patients who relapse after at least two prior LoT. Patient selection is crucial to optimize its risk/benefit ratio and fully harness its curative potential. This includes assessing eligibility criteria, caregiver availability, and potential adherence to therapy. Identifying CAR‐T candidates should begin as early as second‐line therapy, particularly in high‐risk patients, to choose treatments that won't negatively affect CAR‐T efficacy and ensure timely referral to CAR‐T centers. Hematology centers managing FL patients should implement dedicated teams to provide educational and psychological support to patients and caregivers.

## Conflicts of Interest

L.A. received honoraria from EUSA Pharma, Novartis, served on advisory boards and consultations from Roche, incite, EUSA Pharma, Kite/Gilead, Novartis, Morphosys. P.C. declares the following conflict of interest: speaker and advisory board for AbbVie, Amgen, BeiGene, BMS, Daiichi Sankyo, Eli Lilly, Gilead/Kite, GSK, Incyte, Janssen, Jazz Pharma, Novartis, Pfizer, Roche, Sanofi, SOBI, Takeda. M.M. declares the following conflict of interest: advisory board with Novartis, Roche, Astra Zeneca, Sanofi, Menarini, Gilead. L.R. declares the following conflict of interest: speakers bureau for Gilead, Novartis, Sandoz, Abbvie, Servier, Celgene, Janssen, Incyte; advisory board for Gilead, Novartis, Sandoz, Abbvie, Janssen, Incyte, Takeda, AstraZeneca, Eli Lilly. P.L.Z. declares the following conflict of interest: consultant activity for MSD, Takeda, Recordati, Novartis; speakers bureau for Sobi, Kite‐Gilead, Janssen, BMS, MSD, AstraZeneca, Takeda, Roche, Recordati, Kyowa Kirin, Novartis, Incyte, BeiGene; advisory board for Sobi, Kite‐Gilead, Janssen, BMS, MSD, AstraZeneca, Takeda, Roche, Recordati, Kyowa Kirin, Novartis, ADC Therapeutics, Incyte, BeiGene. M.L. declares in the last 5 years the following relationships in terms of consultancy, participation to advisory boards, invitation to scientific meetings, institutional research support and contracts with: AbbVie, Acerta, Amgen, ADC Therapeutics, BeiGene, Celgene/BMS, Eusapharma, GSKI, Gentili, Gilead/Kite, Novartis, Incyte J&J, Jazz, Lilly, Regeneron, Roche, Sandoz. S.L. acted as consultant for Roche, Incyte, BMS, Kite, Novartis, Regenron, Beigene, Sobi.

## Peer Review

The peer review history for this article is available at https://www.webofscience.com/api/gateway/wos/peer-review/10.1002/hon.70125.

## Supporting information


Supporting Information S1

## Data Availability

The data that support the findings of this study are available from the corresponding author upon reasonable request.

## References

[1] J. R. Cerhan , “Epidemiology of Follicular Lymphoma,” Hematology‐Oncology Clinics of North America 34, no. 4 (August 2020): 631–646: Epub 2020 May 5. PMID: 32586570; PMCID: PMC7323888, 10.1016/j.hoc.2020.02.001. 32586570 PMC7323888

[2] S. H. Swerdlow , E. Campo , S. A. Pileri , et al., “The 2016 Revision of the World Health Organization Classification of Lymphoid Neoplasms,” Blood 127, no. 20 (May 2016): 2375–2390: Epub 2016 Mar 15. PMID: 26980727; PMCID: PMC4874220, 10.1182/blood-2016-01-643569. 26980727 PMC4874220

[3] L. R. Teras , C. E. DeSantis , J. R. Cerhan , L. M. Morton , A. Jemal , and C. R. Flowers , “2016 US Lymphoid Malignancy Statistics by World Health Organization Subtypes,” CA: A Cancer Journal for Clinicians 66, no. 6 (November 2016): 443–459: Epub 2016 Sep 12. PMID: 27618563, 10.3322/caac.21357. 27618563

[4] U. Testa , F. D'Alò , E. Pelosi , G. Castelli , and G. Leone , “CAR‐T Cell Therapy for Follicular Lymphomas,” Mediterranean Journal of Hematology and Infectious Diseases 16, no. 1 (January 2024): e2024012: PMID: 38223488; PMCID: PMC10786124, 10.4084/MJHID.2024.012. 38223488 PMC10786124

[5] P. L. Zinzani , J. Muñoz , and J. Trotman , “Current and Future Therapies for Follicular Lymphoma,” Experimental Hematology & Oncology 13, no. 1 (August 2024): 87: PMID: 39175100; PMCID: PMC11340193, 10.1186/s40164-024-00551-1. 39175100 PMC11340193

[6] C. Sarkozy , M. J. Maurer , B. K. Link , et al., “Cause of Death in Follicular Lymphoma in the First Decade of the Rituximab Era: A Pooled Analysis of French and US Cohorts,” Journal of Clinical Oncology 37, no. 2 (January 2019): 144–152: Epub 2018 Nov 27. PMID: 30481079; PMCID: PMC6366812, 10.1200/JCO.18.00400. 30481079 PMC6366812

[7] G. Barosi , A. Carella , M. Lazzarino , et al., “Management of Nodal Indolent (Non Marginal‐Zone) Non‐Hodgkin's Lymphomas: Practice Guidelines From the Italian Society of Hematology, Italian Society of Experimental Hematology and Italian Group for Bone Marrow Transplantation,” Haematologica 90, no. 9 (September 2005): 1236–1257: PMID: 16154848. 16154848

[8] P. L. Zinzani , M. Marchetti , A. Billio , et al., and Expert Panel of the Italian Society of Hematology , “SIE, SIES, GITMO Revised Guidelines for the Management of Follicular Lymphoma,” American Journal of Hematology 88, no. 3 (March 2013): 185–192: Epub 2013 Jan 22. PMID: 23339086, 10.1002/ajh.23372. 23339086

[9] J. L. Brady , M. S. Binkley , C. Hajj , et al., “Definitive Radiotherapy for Localized Follicular Lymphoma Staged by 18F‐FDG PET‐CT: A Collaborative Study by ILROG,” Blood 133, no. 3 (January 2019): 237–245: Epub 2018 Nov 16. Erratum in: Blood. 2019 Jul 18. PMID: 30446493;134(3):331, 10.1182/blood-2018-04-843540. 30446493

[10] A. Pulsoni , S. Ferrero , M. E. Tosti , et al., “Local Radiotherapy and Measurable Residual Disease‐Driven Immunotherapy in Patients With Early‐Stage Follicular Lymphoma (FIL MIRO): Final Results of a Prospective, Multicentre, Phase 2 Trial,” Lancet Haematology 11, no. 7 (July 2024): e499–e509: PMID: 38937025, 10.1016/S2352-3026(24)00143-1. 38937025

[11] Y. Wang , S. Zhou , X. Qi , et al., “Efficacy of Front‐Line Immunochemotherapy for Follicular Lymphoma: A Network Meta‐Analysis of Randomized Controlled Trials,” Blood Cancer Journal 12, no. 1 (January 2022): 1: PMID: 34987165; PMCID: PMC8728708, 10.1038/s41408-021-00598-x. 34987165 PMC8728708

[12] M. Dreyling , M. Ghielmini , S. Rule , et al., and ESMO Guidelines Committee , “Electronic Address: Clinicalguidelines@esmo.Org. Newly Diagnosed and Relapsed Follicular Lymphoma: ESMO Clinical Practice Guidelines for Diagnosis, Treatment and Follow‐Up,” Annals of Oncology 32, no. 3 (March 2021): 298–308: Epub 2020 Nov 26. PMID: 33249059, 10.1016/j.annonc.2020.11.008. 33249059

[13] G. Salles , J. F. Seymour , F. Offner , et al., “Rituximab Maintenance for 2 Years in Patients With High Tumour Burden Follicular Lymphoma Responding to Rituximab Plus Chemotherapy (PRIMA): A Phase 3, Randomised Controlled Trial,” Lancet 377, no. 9759 (January 2011): 42–51: Epub 2010 Dec 20. Erratum in: Lancet. 2011 Apr 2;377(9772):1154. PMID: 21176949, 10.1016/S0140-6736(10)62175-7. 21176949

[14] M. Federico , S. Luminari , A. Dondi , et al., “R‐CVP versus R‐CHOP versus R‐FM for the Initial Treatment of Patients With Advanced‐Stage Follicular Lymphoma: Results of the FOLL05 Trial Conducted by the Fondazione Italiana Linfomi,” Journal of Clinical Oncology 31, no. 12 (April 2013): 1506–1513: Epub 2013 Mar 25. Erratum in: J Clin Oncol. 2014 Apr 1;32(10):1095. Dosage error in article text. PMID: 23530110, 10.1200/JCO.2012.45.0866. 23530110

[15] S. Luminari , A. Ferrari , M. Manni , et al., “Long‐Term Results of the FOLL05 Trial Comparing R‐CVP versus R‐CHOP versus R‐FM for the Initial Treatment of Patients With Advanced‐Stage Symptomatic Follicular Lymphoma,” Journal of Clinical Oncology 36, no. 7 (March 2018): 689–696: Epub 2017 Nov 2. PMID: 29095677, 10.1200/JCO.2017.74.1652. 29095677

[16] M. J. Rummel , N. Niederle , G. Maschmeyer , et al., “Bendamustine Plus Rituximab versus CHOP Plus Rituximab as First‐Line Treatment for Patients With Indolent and Mantle‐Cell Lymphomas: An Open‐Label, Multicentre, Randomised, Phase 3 Non‐Inferiority Trial,” Lancet 381, no. 9873 (2013): 1203–1210: PMID: 23433739, 10.1016/s0140-6736(12)61763-2. 23433739

[17] I. W. Flinn , R. van der Jagt , B. S. Kahl , et al., “Randomized Trial of Bendamustine‐Rituximab or R‐CHOP/R‐CVP in First‐Line Treatment of Indolent NHL or MCL: The BRIGHT Study,” Blood 123, no. 19 (May 2014): 2944–2952: Epub 2014 Mar 3. PMID: 24591201; PMCID: PMC4260975, 10.1182/blood-2013-11-531327. 24591201 PMC4260975

[18] F. Morschhauser , N. H. Fowler , P. Feugier , et al., “RELEVANCE Trial Investigators. Rituximab Plus Lenalidomide in Advanced Untreated Follicular Lymphoma,” New England Journal of Medicine 379, no. 10 (September 2018): 934–947: PMID: 30184451; PMCID: PMC11003525, 10.1056/NEJMoa1805104. 30184451 PMC11003525

[19] R. Marcus , A. Davies , K. Ando , et al., “Obinutuzumab for the First‐Line Treatment of Follicular Lymphoma,” New England Journal of Medicine 377, no. 14 (October 2017): 1331–1344: PMID: 28976863, 10.1056/NEJMoa1614598. 28976863

[20] W. Hiddemann , A. M. Barbui , M. A. Canales , et al., “Immunochemotherapy With Obinutuzumab or Rituximab for Previously Untreated Follicular Lymphoma in the GALLIUM Study: Influence of Chemotherapy on Efficacy and Safety,” Journal of Clinical Oncology 36, no. 23 (August 2018): 2395–2404: Epub 2018 Jun 1. PMID: 29856692, 10.1200/JCO.2017.76.8960. 29856692

[21] C. Casulo , J. G. Dixon , J. Le‐Rademacher , et al., “Validation of POD24 as a Robust Early Clinical End Point of Poor Survival in FL From 5225 Patients on 13 Clinical Trials,” Blood 139, no. 11 (March 2022): 1684–1693: PMID: 34614146; PMCID: PMC9974165, 10.1182/blood.2020010263. 34614146 PMC9974165

[22] A. D. Zelenetz , L. I. Gordon , J. S. Abramson , et al., “NCCN Guidelines® Insights: B‐Cell Lymphomas, Version 6.2023,” Journal of the National Comprehensive Cancer Network 21, no. 11 (November 2023): 1118–1131, 10.6004/jnccn.2023.0057. 37935098

[23] N. H. Fowler , J. C. Chavez , P. A. Riedell , “Moving T‐Cell Therapies Into the Standard of Care for Patients With Relapsed or Refractory Follicular Lymphoma: A Review,” Targeted Oncology 19, no. 4 (July 2024): 495–510. Epub 2024 Jun 19. Erratum in: Target Oncol. 2024 Sep;19(5):817. doi: 10.1007/s11523‐024‐01085‐6. PMID: 38896212; PMCID: PMC11271334 38896212 10.1007/s11523-024-01070-z PMC11271334

[24] C. Casulo , J. W. Friedberg , K. W. Ahn , et al., “Autologous Transplantation in Follicular Lymphoma With Early Therapy Failure: A National LymphoCare Study and Center for International Blood and Marrow Transplant Research Analysis,” Biology of Blood and Marrow Transplantation 24, no. 6 (June 2018): 1163–1171: Epub 2017 Dec 11. PMID: 29242111; PMCID: PMC5993598, 10.1016/j.bbmt.2017.12.771. 29242111 PMC5993598

[25] M. Ladetto , R. Tavarozzi , M. Zanni , et al., “Radioimmunotherapy versus Autologous Hematopoietic Stem Cell Transplantation in Relapsed/Refractory Follicular Lymphoma: A Fondazione Italiana Linfomi Multicenter, Randomized, Phase III Trial,” Annals of Oncology 35, no. 1 (January 2024): 118–129: Epub 2023 Nov 3. PMID: 37922989, 10.1016/j.annonc.2023.10.095. 37922989

[26] F. Morschhauser , H. Tilly , A. Chaidos , et al., “Tazemetostat for Patients With Relapsed or Refractory Follicular Lymphoma: An Open‐Label, Single‐Arm, Multicentre, Phase 2 Trial,” Lancet Oncology 21, no. 11 (November 2020): 1433–1442: Epub 2020 Oct 6. PMID: 33035457; PMCID: PMC8427481, 10.1016/S1470-2045(20)30441-1. 33035457 PMC8427481

[27] P. L. Zinzani , J. Mayer , C. R. Flowers , et al., “ROSEWOOD: A Phase II Randomized Study of Zanubrutinib Plus Obinutuzumab Versus Obinutuzumab Monotherapy in Patients With Relapsed or Refractory Follicular Lymphoma,” Journal of Clinical Oncology 41, no. 33 (November 2023): 5107–5117: Epub 2023 Jul 28. PMID: 37506346, 10.1200/JCO.23.00775. 37506346

[28] C. L. Batlevi , F. Sha , A. Alperovich , et al., “Follicular Lymphoma in the Modern Era: Survival, Treatment Outcomes, and Identification of High‐Risk Subgroups,” Blood Cancer Journal 10, no. 7 (July 2020): 74: PMID: 32678074; PMCID: PMC7366724, 10.1038/s41408-020-00340-z. 32678074 PMC7366724

[29] A. Rivas‐Delgado , L. Magnano , M. Moreno‐Velázquez , et al., “Response Duration and Survival Shorten After Each Relapse in Patients With Follicular Lymphoma Treated in the Rituximab Era,” British Journal of Haematology 184, no. 5 (March 2019): 753–759: Epub 2018 Dec 4. PMID: 30515755, 10.1111/bjh.15708. 30515755

[30] N. H. Fowler , M. Dickinson , M. Dreyling , et al., “Tisagenlecleucel in Adult Relapsed or Refractory Follicular Lymphoma: The Phase 2 ELARA Trial,” Nature Medicine 28, no. 2 (February 2022): 325–332: Epub 2021 Dec 17. PMID: 34921238, 10.1038/s41591-021-01622-0. 34921238

[31] B. Yaniv , B. Tanenbaum , V. Kazakova , and S. A. Patel , “Translational Insights into the Genetics and Immunobiology of Relapsed/Refractory Follicular Lymphoma,” Leukemia Research 142 (May 2024): 107519: Epub ahead of print. PMID: 38761562, 10.1016/j.leukres.2024.107519. 38761562

[32] C. A. Jacobson , J. C. Chavez , A. R. Sehgal , et al., “Axicabtagene Ciloleucel in Relapsed or Refractory Indolent Non‐Hodgkin Lymphoma (ZUMA‐5): A Single‐Arm, Multicentre, Phase 2 Trial,” Lancet Oncology 23, no. 1 (January 2022): 91–103: Epub 2021 Dec 8. PMID: 34895487, 10.1016/S1470-2045(21)00591-X. 34895487

[33] P. Ghione , M. L. Palomba , A. R. Patel , et al., “Comparative Effectiveness of ZUMA‐5 (Axi‐cel) vs SCHOLAR‐5 External Control in Relapsed/refractory Follicular Lymphoma,” Blood 140, no. 8 (August 2022): 851–860: PMID: 35679476; PMCID: PMC9412012, 10.1182/blood.2021014375. 35679476 PMC9412012

[34] S. S. Neelapu , J. C. Chavez , A. R. Sehgal , et al., “Three‐Year Follow‐Up Analysis of Axicabtagene Ciloleucel in Relapsed/Refractory Indolent Non‐Hodgkin Lymphoma (ZUMA‐5),” Blood 143, no. 6 (February 2024): 496–506: PMID: 37879047; PMCID: PMC10934297, 10.1182/blood.2023021243. 37879047 PMC10934297

[35] M. Dreyling , N. H. Fowler , M. Dickinson , et al., “Durable Response After Tisagenlecleucel in Adults With Relapsed/refractory Follicular Lymphoma: ELARA Trial Update,” Blood 143, no. 17 (April 2024): 1713–1725: PMID: 38194692; PMCID: PMC11103095, 10.1182/blood.2023021567. 38194692 PMC11103095

[36] F. Morschhauser , S. Dahiya , M. L Palomba , et al., “Lisocabtagene Maraleucel in Follicular Lymphoma: The Phase 2 TRANSCEND FL Study,” Nature Medicine 30, no. 8 (August 2024): 2199–2207. Epub 2024 Jun 3. Erratum in: Nat Med. 2024 Aug;30(8):2374. doi: 10.1038/s41591‐024‐03175‐4. PMID: 38830991; PMCID: PMC11333271 10.1038/s41591-024-02986-9 PMC11333271 38830991

[37] S. J. Schuster , N. L. Bartlett , S. Assouline , et al., “Mosunetuzumab Induces Complete Remissions in Poor Prognosis Non‐Hodgkin Lymphoma Patients, Including Those Who Are Resistant to or Relapsing After Chimeric Antigen Receptor T‐Cell (CART) Therapies, and is Active in Treatment through Multiple Lines,” supplement, Blood 134, no. S1 (2019): 6, 10.1182/blood-2019-123742.31273004

[38] L. E. Budde , L. H. Sehn , M. Matasar , et al., “Safety and Efficacy of Mosunetuzumab, a Bispecific Antibody, in Patients With Relapsed or Refractory Follicular Lymphoma: A Single‐Arm, Multicentre, Phase 2 Study,” Lancet Oncology 23, no. 8 (August 2022): 1055–1065: Epub 2022 Jul 5. PMID: 35803286, 10.1016/S1470-2045(22)00335-7. 35803286

[39] M. Hutchings , R. Mous , M. R. Clausen , et al., “Dose Escalation of Subcutaneous Epcoritamab in Patients With Relapsed or Refractory B‐Cell Non‐Hodgkin Lymphoma: An Open‐Label, Phase 1/2 Study,” Lancet 398, no. 10306 (2021): 1157–1169, 10.1016/s0140-6736(21)00889-8. 34508654

[40] R. Bannerji , J. E. Arnason , R. H. Advani , et al., “Odronextamab, a Human CD20×CD3 Bispecific Antibody in Patients With CD20‐Positive B‐Cell Malignancies (ELM‐1): Results From the Relapsed or Refractory Non‐Hodgkin Lymphoma Cohort in a Single‐Arm Multicentre, Phase 1 Trial,” Lancet Haematology 9, no. 5 (2022): e327–e339, 10.1016/s2352-3026(22)00072-2. 35366963 PMC10681157

[41] J. Gao , S. Dahiya , and S. A. Patel , “Challenges and Solutions to Superior Chimeric Antigen Receptor‐T Design and Deployment for B‐Cell Lymphomas,” British Journal of Haematology 203, no. 2 (October 2023): 161–168: Epub 2023 Jul 24. PMID: 37488074; PMCID: PMC10913150, 10.1111/bjh.19001. 37488074 PMC10913150

[42] G. Iacoboni , V. Navarro , A. Á Martín‐López , et al., “Recent Bendamustine Treatment Before Apheresis Has a Negative Impact on Outcomes in Patients With Large B‐Cell Lymphoma Receiving Chimeric Antigen Receptor T‐Cell Therapy,” Journal of Clinical Oncology 42, no. 2 (January 2024): 205–217: Epub 2023 Oct 24. PMID: 37874957, 10.1200/JCO.23.01097. 37874957

[43] T. Lotfi , A. Hajizadeh , L. Moja , et al., “A Taxonomy and Framework for Identifying and Developing Actionable Statements in Guidelines Suggests Avoiding Informal Recommendations,” Journal of Clinical Epidemiology 141 (January 2022): 161–171: Epub 2021 Sep 23. PMID: 34562579, 10.1016/j.jclinepi.2021.09.028. 34562579

[44] J. Jones and D. Hunter , “Consensus Methods for Medical and Health Services Research,” BMJ 311, no. 7001 (August 1995): 376–380: PMID: 7640549; PMCID: PMC2550437, 10.1136/bmj.311.7001.376. 7640549 PMC2550437

[45] K. Fitch , S. J. Bernstein , M. Dolores Aguilar , et al., The RAND/UCLA Appropriateness Method User's Manual (RAND Corporation, 2001), https://www.rand.org/pubs/monograph_reports/MR1269.html.

[46] S. Kanters , G. Ball , B. Kahl , et al., “Clinical Outcomes in Patients Relapsed/refractory After ≥ 2 Prior Lines of Therapy for Follicular Lymphoma: A Systematic Literature Review and Meta‐Analysis,” BMC Cancer 23, no. 1 (January 2023): 74: PMID: 36690960; PMCID: PMC9869623, 10.1186/s12885-023-10546-6. 36690960 PMC9869623

[47] C. Casulo , M. Byrtek , K. L. Dawson , et al., “Early Relapse of Follicular Lymphoma After Rituximab Plus Cyclophosphamide, Doxorubicin, Vincristine, and Prednisone Defines Patients at High Risk for Death: An Analysis From the National LymphoCare Study,” Journal of Clinical Oncology 33, no. 23 (August 2015): 2516–2522. Epub 2015 Jun 29. Erratum in: J Clin Oncol. 2016 Apr 20;34(12):1430. Erratum in: J Clin Oncol. 2016 Apr 20;34(12):1430. doi: 10.1200/JCO.2016.67.4879. PMID: 26124482; PMCID: PMC4879714 26124482 10.1200/JCO.2014.59.7534 PMC4879714

[48] C. Sortais , A. Lok , B. Tessoulin , et al., “Progression of Disease Within 2 Years (POD24) is a Clinically Relevant Endpoint to Identify High‐Risk Follicular Lymphoma Patients in Real Life,” Annals of Hematology 99, no. 7 (July 2020): 1595–1604: Epub 2020 May 16. PMID: 32417940, 10.1007/s00277-020-04025-2. 32417940

[49] C. Jommi , S. Bramanti , M. Pani , A. Ghirardini , and A. Santoro , “CAR T‐Cell Therapies in Italy: Patient Access Barriers and Recommendations for Health System Solutions,” Frontiers in Pharmacology 13 (June 2022): 915342: PMID: 35837293; PMCID: PMC9275825, 10.3389/fphar.2022.915342. 35837293 PMC9275825

[50] M. Iqbal , A. Kumar , P. Dreger , et al., “Clinical Practice Recommendations for Hematopoietic Cell Transplantation and Cellular Therapies in Follicular Lymphoma: A Collaborative Effort on Behalf of the American Society of Transplantation and Cellular Therapy and the European Society of Blood and Marrow Transplantation,” Transplantation and Cellular Therapy 30, no. 9 (July 2024): 832–843: Epub ahead of print. PMID: 38972511, 10.1016/j.jtct.2024.06.025. 38972511

[51] G. Shouse , A. V. Danilov , and A. Artz , “CAR T‐Cell Therapy in the Older Person: Indications and Risks,” Current Oncology Reports 24, no. 9 (September 2022): 1189–1199: Epub 2022 Apr 14. PMID: 35420395, 10.1007/s11912-022-01272-6. 35420395

[52] S. J. Yates , J. F. Cursio , A. Artz , et al., “Optimization of Older Adults by a Geriatric Assessment‐Guided Multidisciplinary Clinic Before CAR T‐Cell Therapy,” Blood Advances 8, no. 14 (July 2024): 3785–3797: PMID: 38810262, 10.1182/bloodadvances.2024012727. 38810262 PMC11298834

[53] A. Hurria , S. Gupta , M. Zauderer , et al., “Developing a Cancer‐Specific Geriatric Assessment: A Feasibility Study,” Cancer 104, no. 9 (November 2005): 1998–2005: PMID: 16206252, 10.1002/cncr.21422. 16206252

[54] L. S. Muffly , M. Boulukos , K. Swanson , et al., “Pilot Study of Comprehensive Geriatric Assessment (CGA) in Allogeneic Transplant: CGA Captures a High Prevalence of Vulnerabilities in Older Transplant Recipients,” Biology of Blood and Marrow Transplantation 19, no. 3 (March 2013): 429–434: Epub 2012 Nov 15. PMID: 23160006, 10.1016/j.bbmt.2012.11.006. 23160006

[55] P. Strati , S. Ahmed , F. Furqan , et al., “Prognostic Impact of Corticosteroids on Efficacy of Chimeric Antigen Receptor T‐Cell Therapy in Large B‐Cell Lymphoma,” Blood 137, no. 23 (June 2021): 3272–3276: PMID: 33534891; PMCID: PMC8351896, 10.1182/blood.2020008865. 33534891 PMC8351896

[56] C. Xie , H. Duan , H. Liu , Y. Wang , Z. Sun , and M. Lan , “Promoting Patient‐Centered Care in CAR‐T Therapy for Hematologic Malignancy: A Qualitative Meta‐Synthesis,” Supportive Care in Cancer 32, no. 9 (August 2024): 591: PMID: 39150486; PMCID: PMC11329598, 10.1007/s00520-024-08799-3. 39150486 PMC11329598

[57] S. Yamshon , C. Gribbin , M. Alhomoud , et al., “Safety and Toxicity Profiles of CAR T Cell Therapy in Non‐Hodgkin Lymphoma: A Systematic Review and Meta‐Analysis,” Clinical Lymphoma, Myeloma and Leukemia 24, no. 6 (June 2024): e235–e256.e2: Epub 2024 Feb 15. PMID: 38582666, 10.1016/j.clml.2024.02.007. 38582666

[58] G. Elgohary , Y. Yang , M. Gergis , D. Yi , and U. Gergis , “Chimeric Antigen Receptor T ‐ Cell Therapy for Large B‐Cell Lymphoma Patients With Central Nervous System Involvement, a Systematic Review and Meta‐Analysis,” Clinical Lymphoma, Myeloma and Leukemia 24, no. 4 (April 2024): e142–e151: Epub 2024 Jan 12. PMID: 38267353, 10.1016/j.clml.2023.12.012. 38267353

[59] D. W. Lee , B. D. Santomasso , F. L. Locke , et al., “ASTCT Consensus Grading for Cytokine Release Syndrome and Neurologic Toxicity Associated With Immune Effector Cells,” Biology of Blood and Marrow Transplantation 25, no. 4 (April 2019): 625–638: Epub 2018 Dec 25. PMID: 30592986, 10.1016/j.bbmt.2018.12.758. 30592986 PMC12180426

[60] I. Yakoub‐Agha , C. Chabannon , P. Bader , et al., “Management of Adults and Children Undergoing Chimeric Antigen Receptor T‐Cell Therapy: Best Practice Recommendations of the European Society for Blood and Marrow Transplantation (EBMT) and the Joint Accreditation Committee of ISCT and EBMT (JACIE),” Haematologica 105, no. 2 (January 2020): 297–316: PMID: 31753925; PMCID: PMC7012497, 10.3324/haematol.2019.229781. 31753925 PMC7012497

[61] K. J. Curran , S. Nikiforow , C. Bachier , et al., “A Robust Quality Infrastructure Is Key to Safe and Effective Delivery of Immune Effector Cells: How FACT‐Finding Can Help,” Blood Advances 8, no. 4 (February 2024): 1053–1061: PMID: 37467016; PMCID: PMC10920101, 10.1182/bloodadvances.2023010401. 37467016 PMC10920101

[62] P. Ghione , M. L. Palomba , H. Ghesquieres , et al., “Treatment Patterns and Outcomes in Relapsed/Refractory Follicular Lymphoma: Results From the International SCHOLAR‐5 Study,” Haematologica 108, no. 3 (March 2023): 822–832: PMID: 36263843; PMCID: PMC9973479, 10.3324/haematol.2022.281421. 36263843 PMC9973479

[63] C. Casulo , M. C. Larson , J. J. Lunde , et al., “Treatment Patterns and Outcomes of Patients With Relapsed or Refractory Follicular Lymphoma Receiving Three or More Lines of Systemic Therapy (LEO CReWE): A Multicentre Cohort Study,” Lancet Haematology 9, no. 4 (April 2022): e289–e300: PMID: 35358443; PMCID: PMC9297334, 10.1016/S2352-3026(22)00033-3. 35358443 PMC9297334

[64] J. S. Abramson , M. L. Palomba , L. I. Gordon , et al., “Lisocabtagene Maraleucel for Patients With Relapsed or Refractory Large B‐Cell Lymphomas (TRANSCEND NHL 001): A Multicentre Seamless Design Study,” Lancet 396, no. 10254 (September 2020): 839–852: Epub 2020 Sep 1. PMID: 32888407, 10.1016/S0140-6736(20)31366-0. 32888407

