# Supplementary material for: A SWOT‐Consensus for CAR‐T in Follicular Lymphoma: Fine Tuning of Patient Journey and Selection
Source: Hematol Oncol. 2025 Sep 23;43(5):e70125. doi: 10.1002/hon.70125 (PMC12456390; doi:10.1002/hon.70125)
Supplement: Supplementary file 1 — Supporting Information S1 [file HON-43-e70125-s001.docx]

**Appendix**

**Figure 1.** Outcome ranking Question1. The board voted on a 7-point Likert scale where 1 was “not very important” and 7 was “very important.”


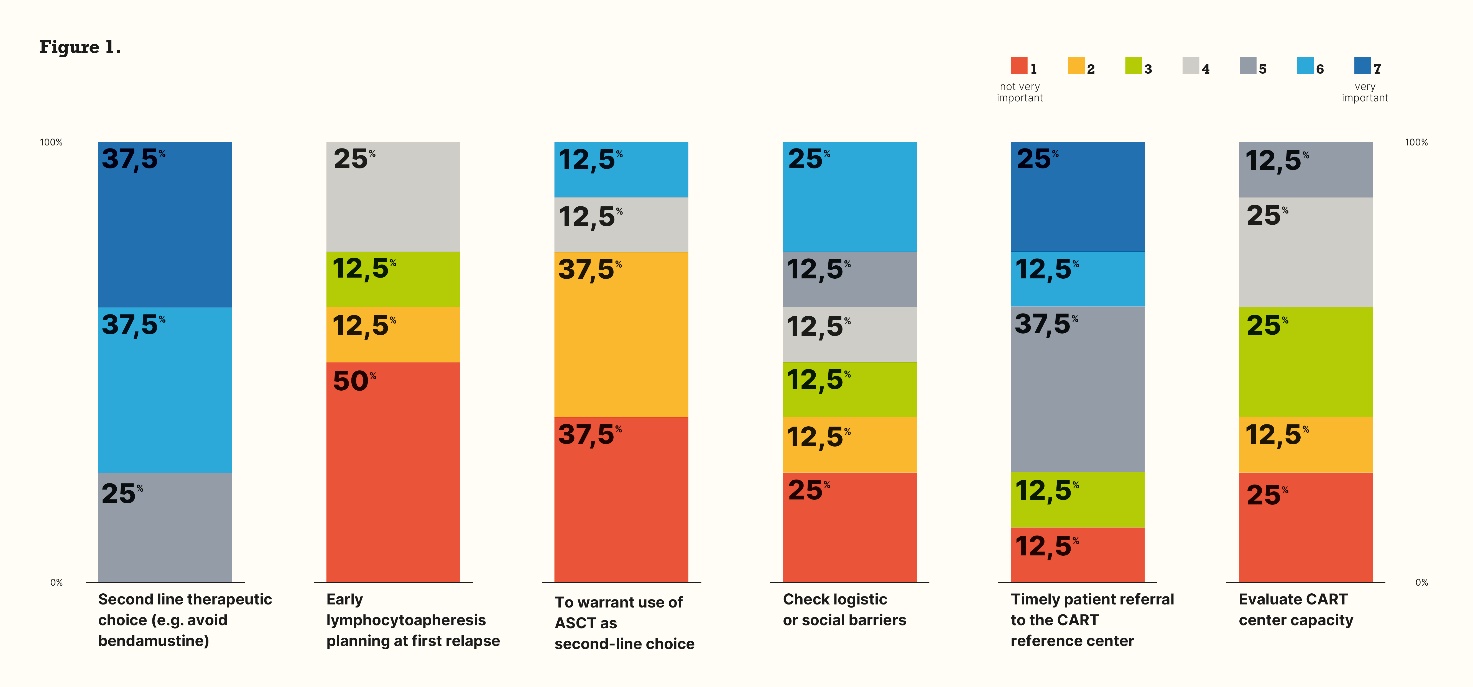


**Figure 2.** Subgroup ranking Question1. The board voted on a 7-point Likert scale where 1 was “not very important” and 7 was “very important.”


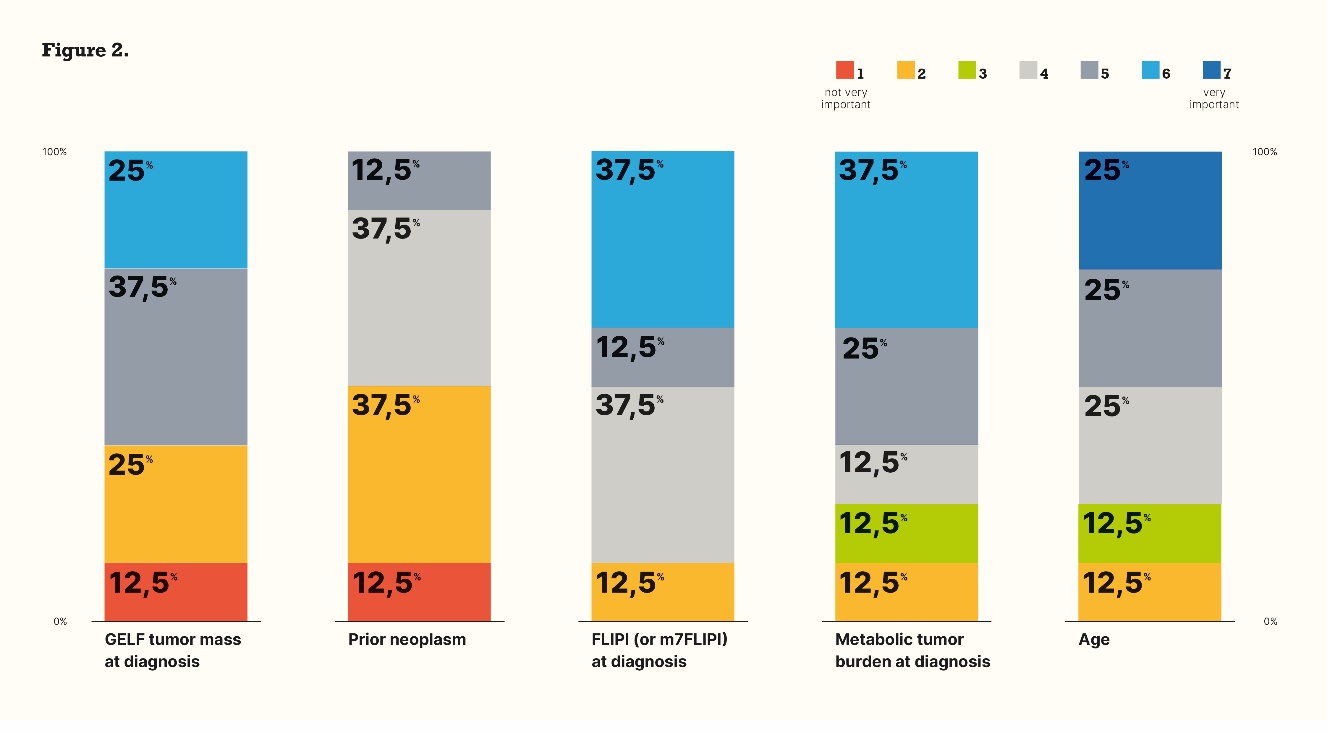


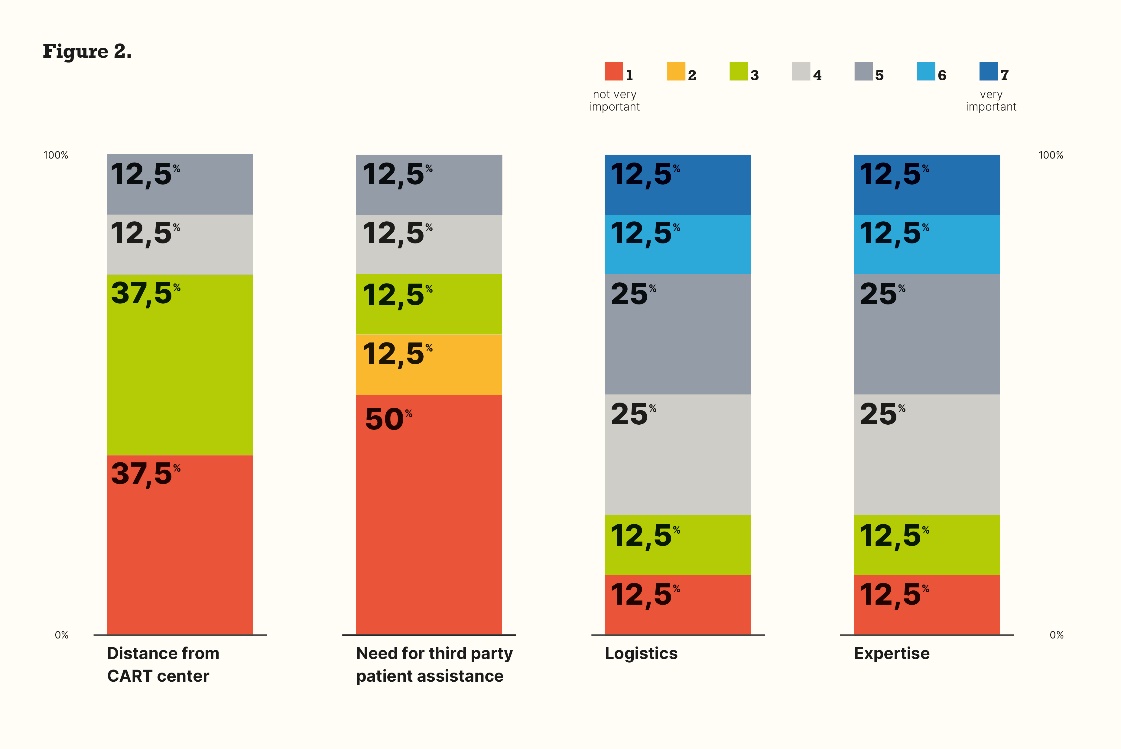


**Figure 3**. Outcome ranking Question2. The board voted on a 7-point Likert scale where 1 was “not very important” and 7 was “very important.”


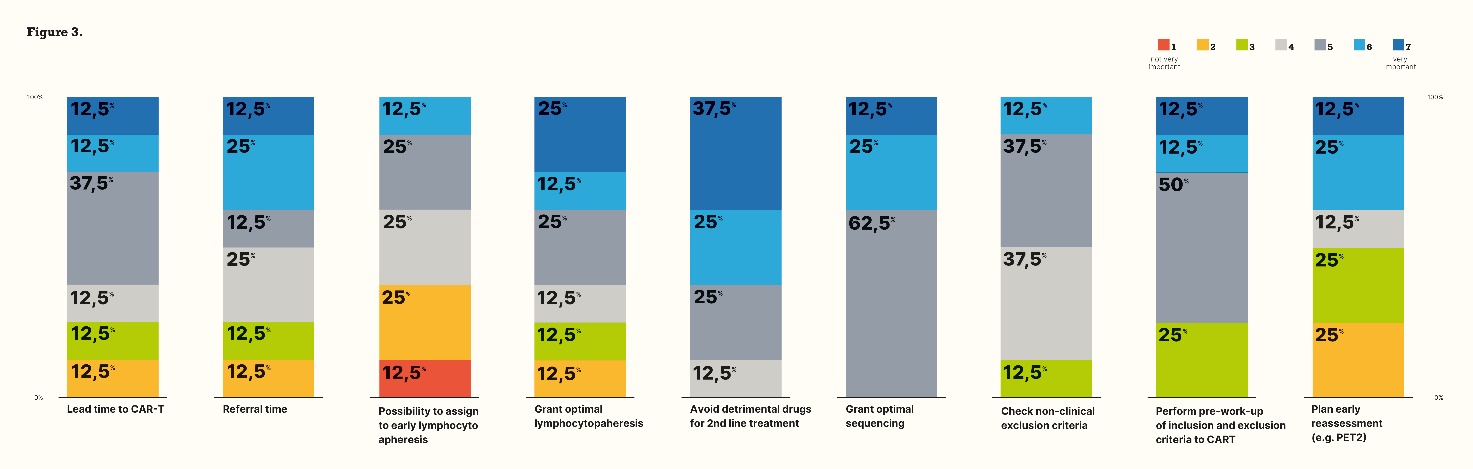


**Figure 4**. Subgroup ranking Question 2. The board voted on a 7-point Likert scale where 1 was “not very important” and 7 was “very important.”


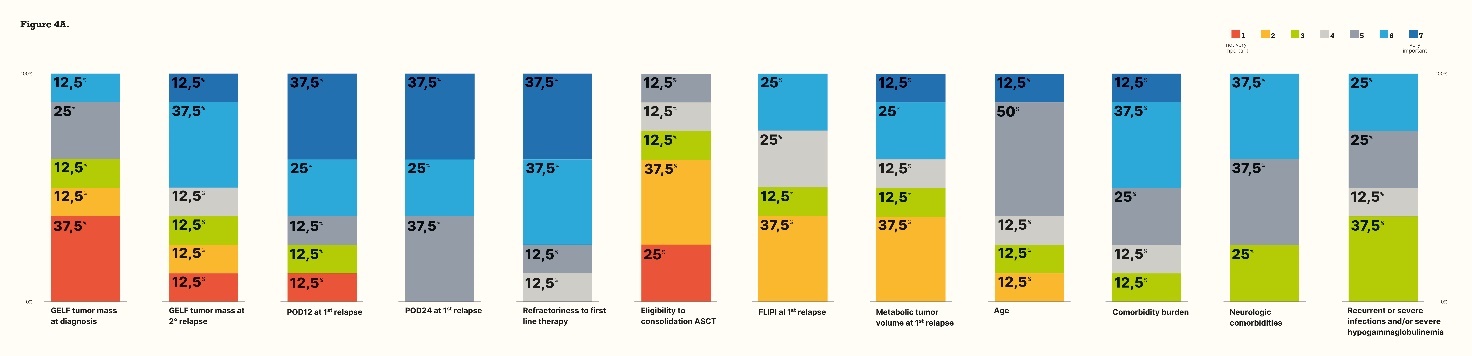


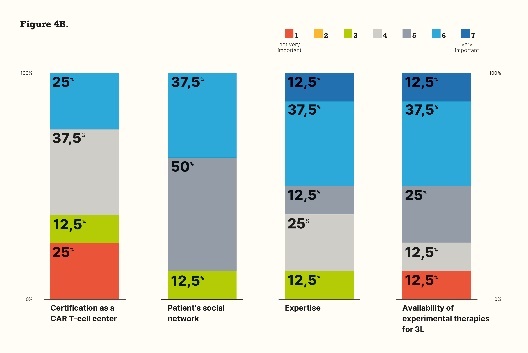


**Figure 5**. Outcome ranking Question3A. The board voted on a 7-point Likert scale where 1 was “not very important” and 7 was “very important.”


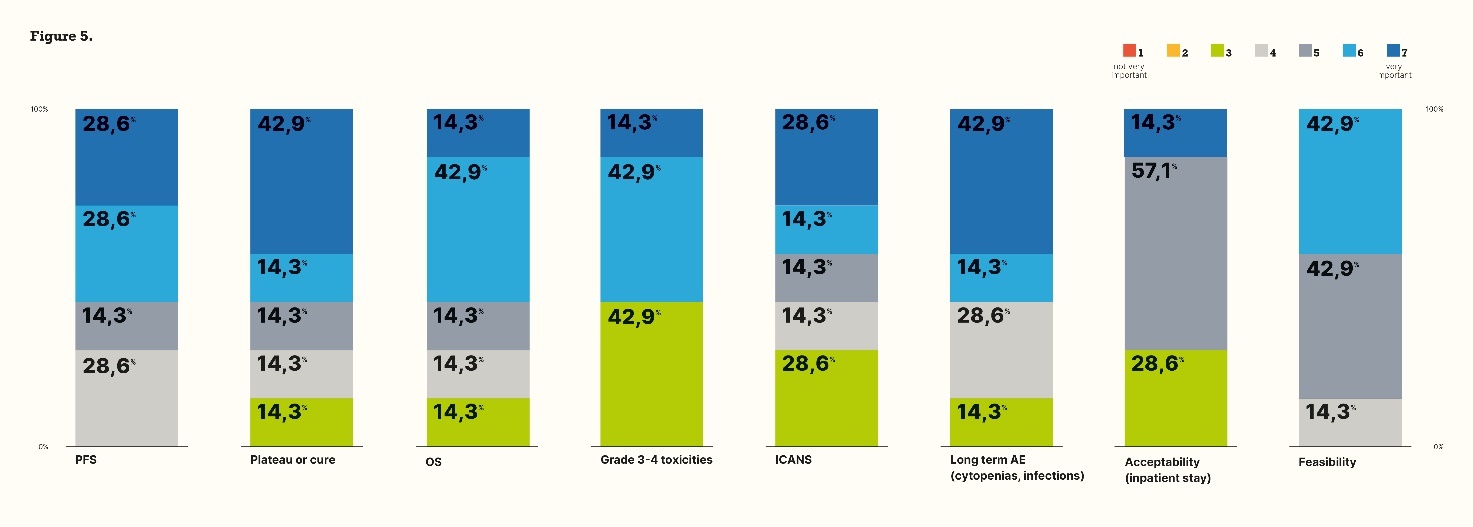


**Figure 6.** Subgroup ranking Question 3A. The board voted on a 7-point Likert scale where 1 was “not very important” and 7 was “very important.”


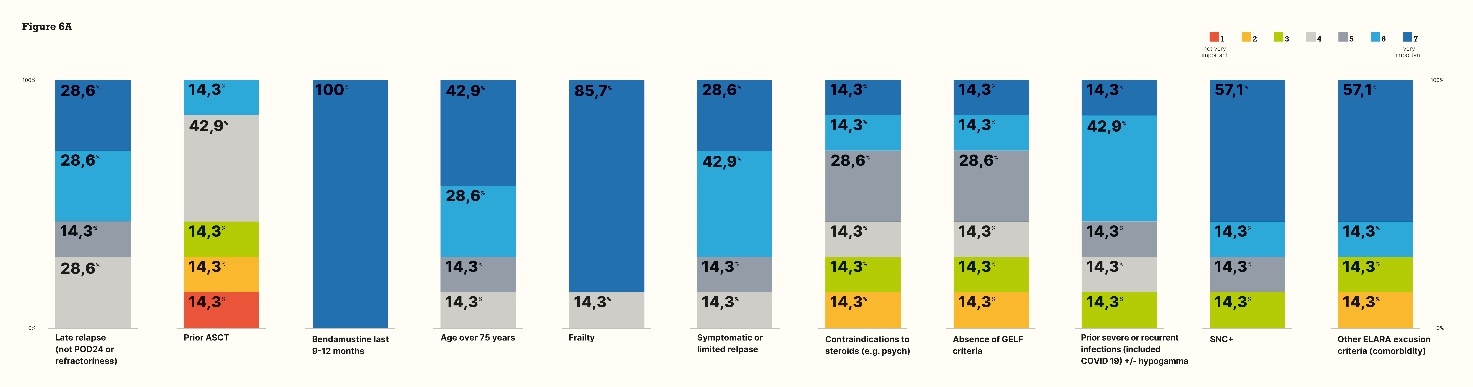


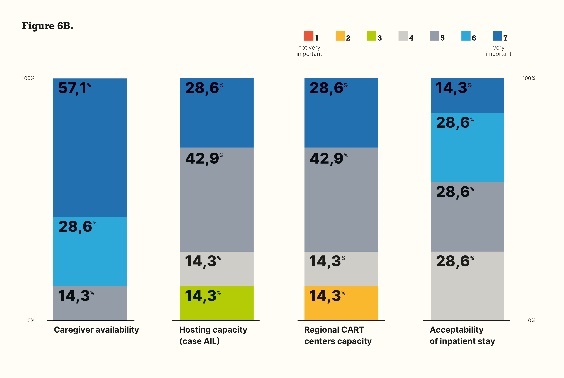


**Figure 7**. Outcome ranking Question 3B. The board voted on a 7-point Likert scale where 1 was “not very important” and 7 was “very important.”


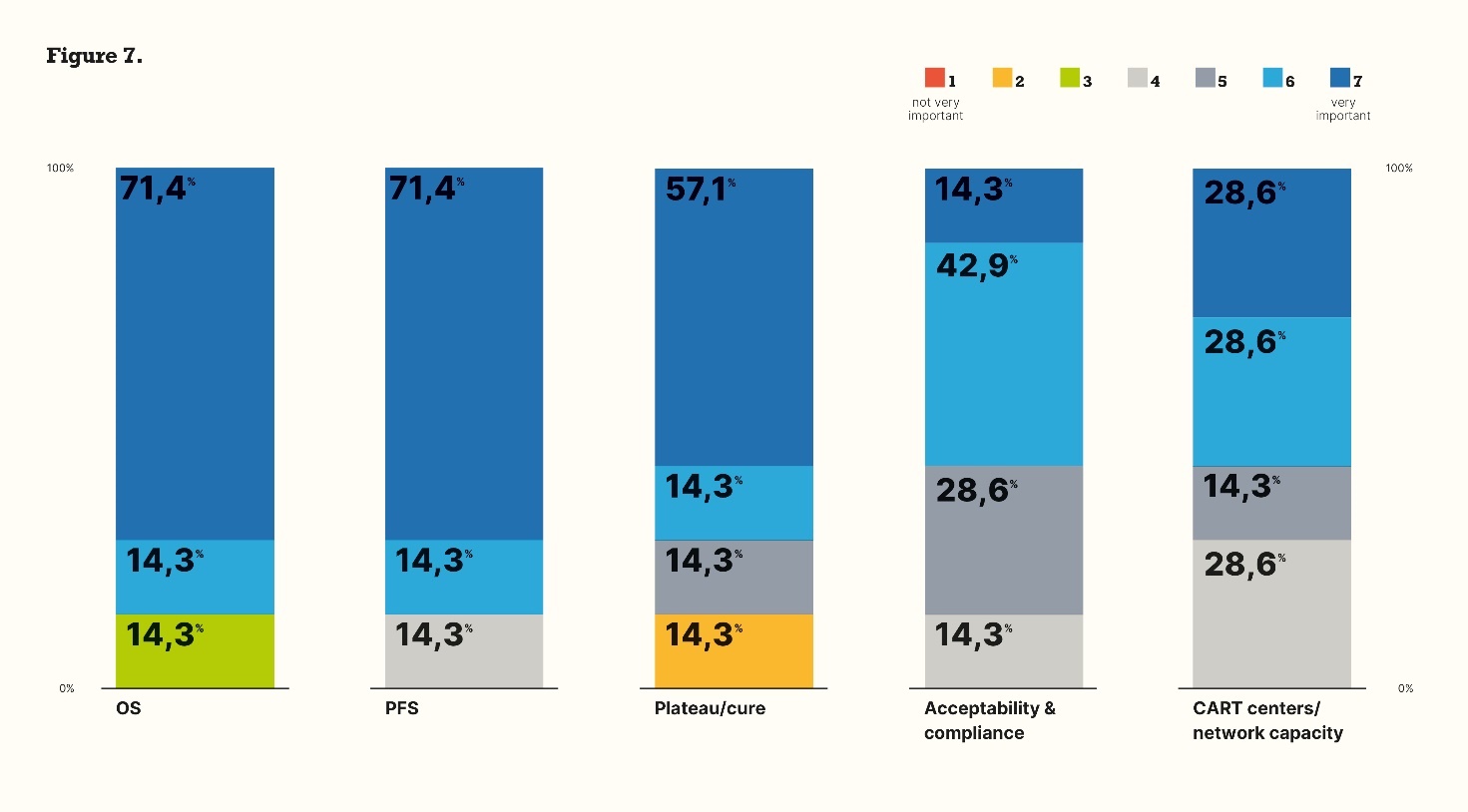


**Figure 8**. Subgroup ranking Question 3B. The board voted on a 7-point Likert scale where 1 was “not very important” and 7 was “very important.”


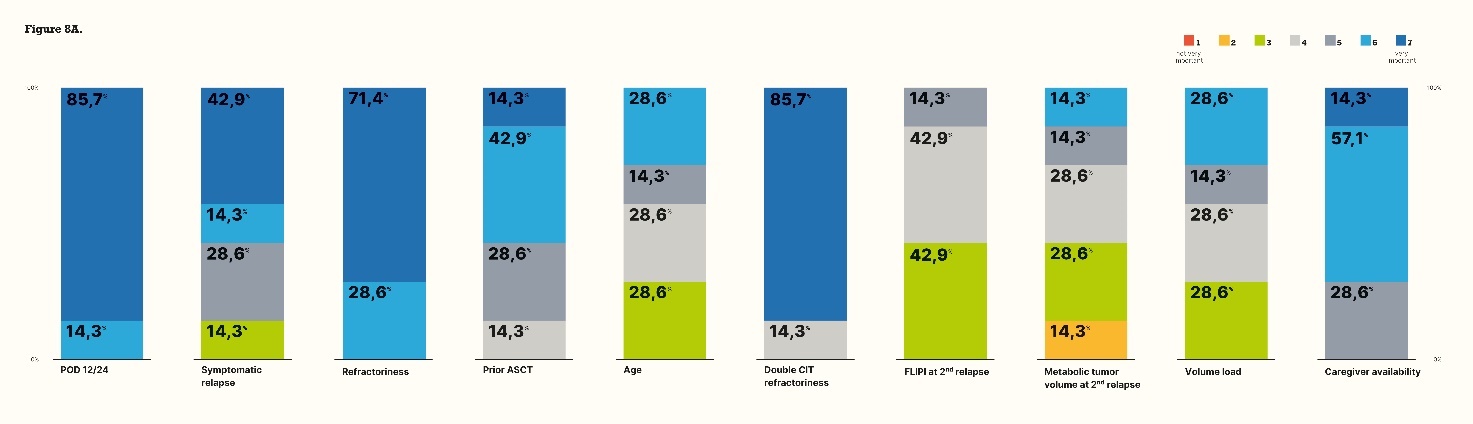


**Table 1.** Whole panel agreement on statement.
